# Hereditary Gingival Fibromatosis and Developmental Anomalies: A Case Report

**DOI:** 10.7759/cureus.24219

**Published:** 2022-04-17

**Authors:** Rhaina A Afonso, Géssica V Godinho, Cristhiane A Silva, Everton J Silva, Luiz E Volpato

**Affiliations:** 1 Department of Dentistry, Hospital de Câncer de Mato Grosso, Cuiabá, BRA; 2 Department of Environment and Health, Universidade de Cuiabá, Cuiabá, BRA; 3 Cuiabá Dental School, Universidade de Cuiabá, Cuiabá, BRA

**Keywords:** autosomal dominant genetic disorder, rare genetic disorder, platelet-rich fibrin, gingivectomy, gingival fibromatosis

## Abstract

Hereditary gingival fibromatosis is the most common genetic form of gingival fibromatosis that develops as a slow, progressive, benign, localized, or generalized enlargement of the keratinized gingiva. It is a genetically heterogeneous disorder transmitted as an autosomal dominant or autosomal recessive trait or appears sporadically. Here, we report a case of a male patient with generalized gingival hyperplasia with great tissue extension to the palatal region, bilateral mandibular torus, bilateral exostosis in the maxillary posterior region, anterior open bite, and diastema in anterior maxilla and mandible teeth. The mucous membranes were healthy and normal colored, with pale pink gums and firm teeth upon palpation. Computed tomography also revealed images suggestive of supernumerary teeth. The patient reported that his mother and a maternal aunt have the same gingival condition. Considering the gingival characteristic, the patient's family history, and the absence of other possible etiological factors of gingival hyperplasia, the diagnostic hypothesis was hereditary gingival fibromatosis. The surgical removal of the enlarged tissue through gingivectomy with internal bevel, osteoplasty, and removal of supernumerary teeth with a subsequent filling of the surgical sites with platelet and leukocyte-rich fibrin membranes in the same surgical time presented good functional and aesthetic results for the young patient with hereditary gingival fibromatosis. It is a viable possibility for clinical management of similar cases.

## Introduction

Gingival fibromatosis is a rare condition characterized by a slow and progressive benign fibrous growth of the gingival tissues, both in the maxilla and in the mandible, with a genetic predisposition in most cases [1]. When gingival fibromatosis has a genetic origin, it can occur in isolation or be part of a syndrome. Its origin can also be acquired, related to the use of specific drugs, mainly phenytoin, cyclosporine, and nifedipine, administered systemically [2]. The prevalence of the condition is low, at 1:175,000, with men and women being equally affected [2].

The mechanism leading to gingival enlargement in hereditary gingival fibromatosis is unknown, but it is known that there is a greater proliferation of subepithelial fibroblasts and a greater synthesis of collagen and fibronectin and, at the same time, a reduction in matrix metalloproteinases (MMPs) responsible for degradation of collagen [3].

Clinically, the affected tissue presents with a pinkish aspect, is firm, painless, and non-hemorrhagic, and covered by a smooth surface distributed symmetrically in the arch. Although gingival enlargement does not directly affect the alveolar bone, gingival edema can favor the accumulation of biofilm, inducing gingivitis, periodontitis, bone resorption, and halitosis [4]. The excess of gingival tissue can result in diastema, tooth displacement, and tooth impaction [5].

The recommended surgical treatment consists of the excision of the excessive gingival tissue associated with strict control of oral hygiene. Many techniques have been used for the excision of enlarged gingival tissues, including external or internal bevel gingivectomy associated with gingivoplasty, apically positioned flap, and surgery using electrocautery or carbon dioxide laser [6].

This article reports the clinical case of a non-syndromic patient with hereditary gingival fibromatosis and its treatment.

## Case presentation

A 24-year-old male patient attended the Oral and Maxillofacial Surgery and Traumatology Outpatient Clinic of the Mato Grosso Cancer Hospital complaining of shame when smiling and pain in the hard palate region when chewing.

**Figure 1:**
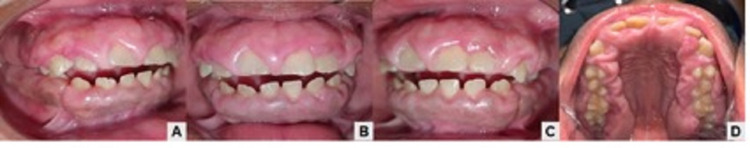
Clinical aspect of the patient showing a large increase in gingival volume with normal color and tooth displacement.

On clinical examination, regular oral hygiene, generalized gingival hyperplasia with great tissue extension in the palatal region, bilateral mandibular torus, bilateral exostosis in the maxillary posterior region, anterior open bite, and diastema in anterior maxilla and mandible teeth were observed. The mucous membranes were healthy and normal colored, with pale pink gums and firm teeth upon palpation (Figure 1). The patient reported that his condition started in his youth around puberty and his mother and a maternal aunt have the same gingival condition.

**Figure 2:**
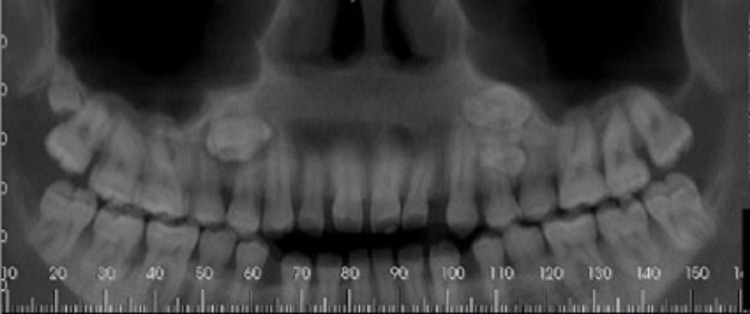
Computed tomography image in panoramic reconstruction showing the presence of four included supernumerary teeth in the region of upper premolars bilaterally and the presence of a fourth molar on the right side.

Computed tomography revealed images suggestive of supernumerary teeth: upper right fourth molar, upper right parapremolar, and upper left parapremolar, with a geminated appearance (Figure 2).

Considering the gingival characteristic, the patient's family history, and the absence of other possible etiological factors of gingival hyperplasia, the diagnostic hypothesis was hereditary gingival fibromatosis.

**Figure 3:**
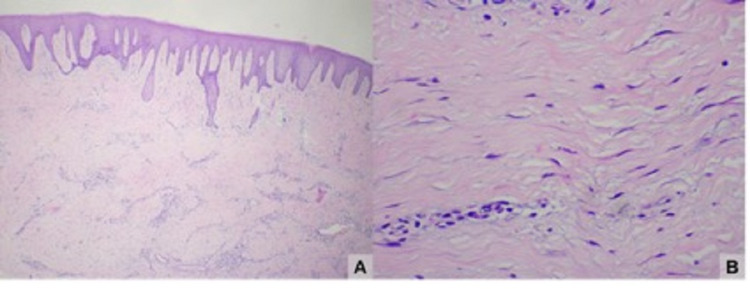
Histological section in (A) 40x magnification showing a stratified squamous-type surface epithelium with long epithelial ridges extending deeply and in (B) 400x magnification showing a dense and hypocellular underlying fibrous connective tissue exhibiting mild inflammation and sometimes cells resembling myofibroblasts.

Under general anesthesia and nasotracheal intubation, gingivectomy and osteotomy were performed to enlarge the clinical crowns of erupted teeth and osteoplasty to regularize vestibular exostosis and extraction of supernumerary teeth and third molars. To assist in bone repair, platelet and leukocyte-rich fibrin membranes (PRF-L) were used in the parapremolar extraction sites. The removed gingival material was sent for anatomopathological examination, whose report consisted of the proliferation of spindle cells arranged in parallel fascicles, associated with a variable amount of collagen. Lesions are usually poorly circumscribed and infiltrate adjacent tissues. Hyperchromatism and cellular pleomorphism may not be observed (Figure 3).

**Figure 4:**
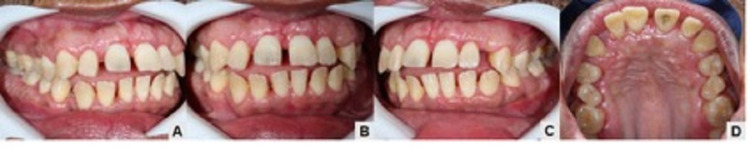
Patient’s clinical appearance on postoperative day 30 showing reestablishment of gingival contours and adequate exposure of dental crowns.

On the 30-day postoperative follow-up, there were significant clinical and functional improvements, with satisfactory healing and the absence of signs of inflammation or infection (Figure 4). The patient reported improvement in chewing and aesthetic satisfaction when compared to the preoperative condition. Instructions on oral hygiene and referral to the general practitioner for necessary restorative treatments and the orthodontist for dental alignment were carried out.

## Discussion

This work reports the clinical case of a young patient with hereditary gingival fibromatosis with significant gingival and bone volume excesses, in addition to the presence of four included supernumerary teeth. It was possible to establish a treatment plan with gingivectomy, followed by vestibular bone plasty, and extraction of the third molars and supernumerary teeth, allowing the aesthetic and functional reestablishment.

Gingival fibromatosis is a generic term used to identify an increase in gingival volume caused by the accumulation of large amounts of collagen, classified as idiopathic, inflammatory, medicinal, or hereditary [7]. The case presented fits the hereditary type, considering the presence of similar cases in the family and the absence of a history of associated diseases and/or use of medications by the patient.

Furthermore, the clinical aspect of gingival fibromatosis differs according to its etiology. Gingival enlargement resulting from hereditary gingival fibromatosis affects, in addition to the attached gingiva and interdental papillae, the marginal gingiva, unlike drug-induced hyperplasia [7]. This clinical characteristic is seen in this case, reinforcing its hereditary origin.

Other conditions that may resemble hereditary gingival fibromatosis, in addition to gingival hyperplasia induced by drugs such as phenytoin, cyclosporine-A, nifedipine, and verapamil, are inflammatory gingival hyperplasia (induced by bacterial biofilm), some metabolic disorders that can also trigger dilated gingival enlargement such as diffuse angiokeratomas of the body (Fabry's disease) and Hurler's syndrome [5], and rare genetic disorders such as craniofacial dysmorphism, Murray-Puretic-Drescher syndrome, Zimmermann-Laband syndrome, nephrocalcinosis syndrome, amelogenesis imperfecta syndrome, which occur with a prevalence of one or less per million population [1].

The mechanism that leads to gingival enlargement in hereditary gingival fibromatosis is unknown [8]. It is known that there is a greater proliferation of subepithelial fibroblasts and a greater synthesis of collagen and fibronectin and, at the same time, a reduction in MMPs responsible for collagen degradation [8].

Hereditary gingival fibromatosis does not regress spontaneously and the treatment of choice is gingivectomy, which can be performed with an internal or external bevel incision [9]. In the present case, we opted for gingivectomy with internal bevel and ostectomy aiming to increase the clinical crown and osteoplasty to regularize the buccal exostosis. Although the gingival enlargement does not directly affect the alveolar bone, in the present case, there was an increase in the buccal bone volume, in which osteoplasty was performed to provide passive adaptation of the gingival tissues. It is noteworthy that most cases do not involve bone but only the gingival tissue [10].

In the present case, we opted to use PRF-L in the parapremolar extraction sites to assist in bone repair and maintenance of bone volume. Platelet aggregate is an alternative biomaterial used in oral surgery to assist in the healing process, especially in areas where bone wear has taken place. Among the types of concentrates, fibrin-rich plasma (FRP) is considered the most similar to the natural clot, as it does not require any type of biochemical substance and is obtained from pure blood. This biomaterial favors the development of a coherent healing matrix without inflammatory excesses [10].

Performing surgery after the eruption of permanent teeth reduces the recurrence rate of gingival fibromatosis [4]. Treatment shall be carried out early because the pathology causes a negative psychological effect on the patient, in addition to functional difficulties. The follow-up must be of short and long term, as relapses may be present. The case presented is currently under one-year postoperative follow-up with no signs of recurrence.

## Conclusions

In the case described, the surgical removal of the enlarged tissue through gingivectomy with internal bevel, osteoplasty, and removal of supernumerary teeth with a subsequent filling of the surgical sites with platelet and leukocyte-rich fibrin membranes in the same surgical time presented good functional and aesthetic results for the young patient with hereditary gingival fibromatosis. It is a viable possibility for clinical management of similar cases.
